# Endoscopic ultrasound-guided portal pressure gradient assessment of acute hemodynamic response to intravenous propranolol

**DOI:** 10.1055/a-2764-4494

**Published:** 2026-01-13

**Authors:** Rafael Romero-Castro, Enrique Silva-Albarellos, Lourdes Grandes-Santamaria, Isabel Carmona-Soria, Victoria Alejandra Jimenez-Garcia, Manuel Rodriguez-Tellez, Ángel Caunedo-Álvarez

**Affiliations:** 1 16582 Virgen Macarena University Hospital, Seville, Spain; 2 222071 Vithas Sevilla Hospital, Castilleja de la Cuesta, Seville, Spain; 3 222064 Hospital San Agustin, Seville, Spain

The development of portal hypertension is a critical hallmark in chronic liver diseases. The hepatic venous pressure gradient (HVPG) is the gold-standard method to diagnose and quantify portal hypertension and the hemodynamic response to drug therapy. An acute hemodynamic response to intravenous propranolol assessed with the HVPG predicts adverse liver-related events 1 . However, the HVPG is not recommended in routine clinical care due to its drawbacks 2 . Non-invasive tests (NITs) are used in daily clinical practice to stratify the risk of clinically significant portal hypertension 2 . Nevertheless, NITs are not recommended for assessing hemodynamic changes in portal hypertension 2 . Moreover, there is a gray-zone where patients affected with metabolic associated liver diseases, obesity or mixed etiologies could be misclassified by NITs 3 . The endoscopic ultrasound-guided portal pressure gradient measurement (EUS-PPGm) has been accurately compared to the HVPG and can directly obtain the real portal vein pressure 4 .

We determine the acute hemodynamic response to intravenous propranolol with EUS-PPGm in a case of a series of four patients ( Table 1 ). The procedure was performed as previously reported, taking into account several tips and tricks to avoid non-reliable results 5 .

**Table:** **Table 1** Data and results.

Patient	Age	Sex	Etiology	Child-Pugh	MELD	Liver stiffness (kPa)	Platelets (g/L)	EUS-PPGm before iv ropranolol	EUS-PPGm after iv propranolol
1	60	Male	Alcohol	B8	12	75	164.000	3 mmHg	Not indicated
2	67	Female	Alcohol	A5	8	19	108.000	3.75 mmHg	Not indicated
3	60	Male	Metabolic	A5	7	29	170.000	10.5 mmHg	4.75 mmHg (55% reduction)
4	65	Female	Alcohol	B9	11	26,5	147.000	10 mmHg	2 mmHg (80% reduction)
Abbreviation: EUS-PPGm, endoscopic ultrasound-guided portal pressure gradient measurement.									

Following baseline EUS-PPGm, 0.15 mg/kg of body weight, propranolol was administered intravenously by continuous infusion in 10 minutes. The second EUS-PPGm was repeated 15 minutes later targeting the same vessels with the same angle of the needle and position of the echoendoscope as in the baseline EUS-PPGm procedure ( Video 1 ).

EUS-guided portal pressure gradient assessment of the acute hemodynamic response to intravenous propranolol.Video 1

**Fig. 1:**
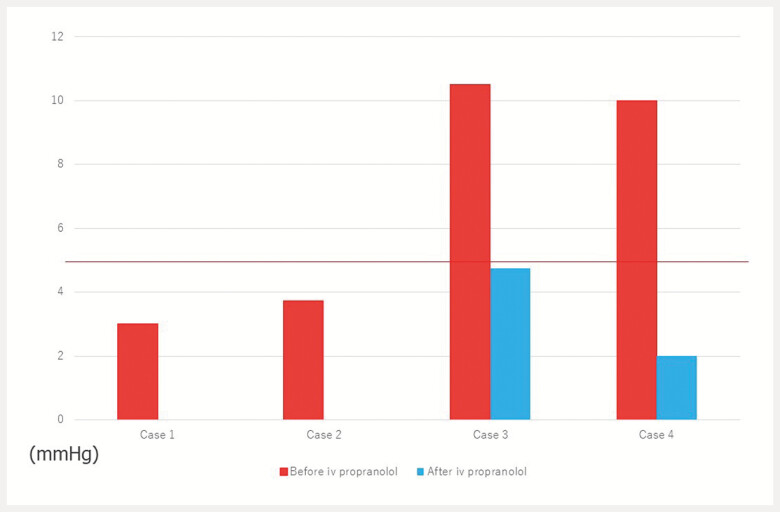
EUS-guided assessment of the portal pressure gradient after intravenous administration of propranolol. EUS, endoscopic ultrasound.

A significant reduction of PPG in two patients ( Fig. 1 ), treated with intravenous propranolol, was observed (10.5 mmHg to 4.75 mmHg [55%] and 10 mmHg to 2 mmHg [80%], respectively). In the other two patients, the first EUS-PPGm was normal. So, a second EUS-PPG measurement was not performed avoiding further unnecessary therapy with beta-blockers. No adverse events were observed.

EUS-PPGm can assess acute hemodynamic changes after intravenous administration of propranolol.

Endoscopy_UCTN_Code_TTT_1AS_2AD
